# Researches on the Motion of the Juices in the Animal Body

**Published:** 1848-08

**Authors:** 


*Researches on the Motion of the Juices in the Animal Body.* By JUSTUS LIEBIG, M. D., Professor of Chemistry in the University of Giessen. Edited from the Manuscript of the Author by WILLIAM GREGORY, M. D., Professor of Chemistry in the University of Edinburgh. 8vo., pp. 109. London, 1848.

We think that Liebig has some reason to be dissatisfied with the anxiety of certain of his friends, and especially of Dr. Gregory, to act as gentlemen ushers to every investigation, crude or trite, into which he may enter; but if he be satisfied with them, we are not so with him; inasmuch as we think such publications as the one before us, and his "Researches on the Chemistry of Food," are better fitted for the pages of a periodical. They certainly—if valuable—will be attainable by a much larger number of individuals than can now read them. It is true they have been reprinted in this country by Professor Horsford, of Cambridge, at a comparatively cheap rate, but still their circulation cannot but be restricted.

The work before us contains the results of Liebig's investigations on endosmosis; but we see nothing or little that is novel in the results at which he has arrived. From these results Dr. Gregory states "the reader will," he trusts, "be satisfied that the motions of the animal juices depend on something more than mere endosmosis or exosmosis; and that the pressure of the atmosphere, as well as its hygrometric state, by influencing the transpiration from the skin and lungs, are [is] essentially concerned in producing these motions." Doubtless, these are important agencies; but they are of limited operation, and have an undue weight assigned to them by Liebig; and so we would say have the forces invoked by Professor Draper as concerned in the motion of the blood. Most physiologists—Dr. Draper properly remarks—have regarded the hydraulic action of the heart as amply sufficient to account for all the phenomena. "It is now," he adds, "on all hands conceded that this organ discharges a very subsidiary duty." Such is not our impression. We admit that numerous sources of motion exist; but we cannot regard the action of that powerful muscular organ which possesses an active power of con traction and dilatation as "very subsidiary" in the circulation.

On the contrary, we esteem the forces in the motion of the juices invoked by Liebig as well as those by Dr. Draper, to be altogether ancillary to the great source of movement.

"From what has gone before, it can hardly be doubted," says the former, "that one of the most important functions of the skin consists in the share which it takes in the motion and distribution of the fluids of the body. The surface of the body of a number of animals consists of a covering or skin serviceable for liquids, from which, when, as in the case of the lungs, it is in contact with the atmosphere, an evaporation of water, according to the hygrometric state and temperature of the air, constantly goes on. If we now keep in mind, that every part of the body has to sustain the pressure of the atmosphere, and that the gaseous fluids and liquids contained in the body oppose to this pressure a perfectly equal resistance, it is clear, that, by the evaporation of the skin and lungs, and in consequence of the absorbent power of the skin for the liquid in contact with it, a difference in the pressure below the surface of the evaporating skin occurs. The external pressure increases, and in an equal degree the pressure from within towards the skin. If now the structure of the cutaneous surface does not permit a diminution of its volume, a compression (in consequence of the loss of liquid by evaporation,) it is obvious that an equalization of this difference in pressure can only take place from within outwards; first from within, and especially from those parts which are in closest contact with the atmosphere, and which offer the least resistance to the action of the external pressure. Hence it follows, that the fluids of the body, in consequence of the cutaneous and pulmonary transpiration, acquire a motion towards the skin and lungs, which must be accelerated by the circulation of the blood." p. 74.

But we must leave those who are anxious to pursue the subject to refer to the translation,—not the clearest, by the way, in language, and a part of this, perhaps, applicable to the author.

